# On the Use of the Elastic Gum Catheter

**Published:** 1813-05

**Authors:** 


*To the Editors of the Medical and Physical Journal.*

GENTLEMEN,

PERMIT me, through the medium of your useful Journal, to be informed by any practitioner of surgery, what success they can derive from the use of the Elastic Gum Catheter, for a length of time, immediately after the operation of lithotomy, which, by a fair trial, I am strongly convinced, would be productive of many beneficial effects.

Among the many celebrated writers on the stone in the bladder, I cannot find such a simple practice introduced; and in patients I have seen undergo the operation, many unpleasant and dangerous symptoms followed by the length of time the parts took to heal, and the urine was often some time before it could be evacuated by its natural canal: this, then, is my reason for proposing the catheter.

In the first instance, I beg leave to say, that wearing the elastic gum catheter, assists greatly in conveying the urine from the bladder, and allows those parts injured by the operation an opportunity of healing much quicker than otherwise they would do; secondly, it prevents the growth of excrescences, and other obstructions in the urethra, which are often known to occur, and must be attended with disagreeable consequences; thirdly, the great facility, in many cases, of introducing this catheter, and the trifling inconveniences attending the constant use of it, render it again worthy the attention of any unprejudiced surgeon.

By giving this little communication a place in your valuable miscellany, you will greatly oblige

Your obedient humble Servant,

